# The underestimated burden of placental schistosomiasis in endemic regions: Findings from a cross-sectional, diagnostic proof-of-concept study on placental schistosomiasis in Gabon

**DOI:** 10.1371/journal.pntd.0014031

**Published:** 2026-03-03

**Authors:** Saskia Dede Davi, Dearie Glory Okwu, Yabo Josiane Honkpehedji, Pia Michelitsch, Lillian Rene Endamne, Jacob Gerstenberg, Josephine Mischlinger, Rella Zoleko-Manego, Paul Alvyn Nguema-Moure, Ayola Akim Adegnika, Egbert Tannich, Stefan Lueth, Martha Holtfreter, Dennis Tappe, Ghyslain Mombo-Ngoma, Michael Ramharter, Benjamin Thomas Schleenvoigt, Johannes Mischlinger

**Affiliations:** 1 Center for Tropical Medicine, Bernhard-Nocht Institute for Tropical Medicine & I. Dept. of Medicine University Medical Center Hamburg-Eppendorf, Hamburg, Germany; 2 German Center for Infection Research, Partner Site Hamburg-Lübeck-Borstel-Riems, Hamburg-Lübeck-Borstel-Riems, Hamburg, Germany; 3 Centre de Recherches Médicales de Lambaréné, Lambaréné, Gabon; 4 Leiden Center of Infection Diseases, Leiden University Medical Center, Leiden, the Netherlands; 5 Department of Internal Medicine, DIAKOVERE Friederikenstift, Hannover, Germany; 6 Institute of Infectious Diseases and Infection Control, Jena University Hospital, Friedrich-Schiller-University, Jena, Germany; 7 Institute for Tropical Medicine, University Hospital Tübingen, Tübingen, Germany; 8 Translational Thematic Unit Malaria, German Center for Infection Research (DZIF), Tübingen, Germany; 9 Bernhard Nocht Institute for Tropical Medicine, Hamburg, Germany; 10 Department of Gastroenterology, Faculty of Health Sciences Brandenburg, Brandenburg Medical School Theodor Fontane, Brandenburg an der Havel, Germany; 11 Department of Gastroenterology, Hepatology and Infectious Diseases, Medical Faculty and University Hospital Duesseldorf, Heinrich-Heine-University Duesseldorf, Duesseldorf, Germany; 12 Department of Implementation Research, Bernhard-Nocht Institute for Tropical Medicine, Hamburg, Germany; George Washington University School of Medicine and Health Sciences, UNITED STATES OF AMERICA

## Abstract

**Background:**

Placental schistosomiasis (PS) is underdiagnosed and may compromise maternal and neonatal health. This study estimated the prevalence of PS in a rural Gabonese population of pregnant women with confirmed *S. haematobium* infection using light microscopy of macerated placental tissue.

**Methods:**

This is a cross-sectional, diagnostic proof-of-concept study which applied an improved placenta maceration technique in real-world conditions to diagnose PS. Performing light microscopic assessment of a single sample of 10 mL urine, we screened pregnant women for *S. haematobium* infection who sought antenatal care in Lambaréné (Gabon) between January 2022 and January 2023. Women positive for *S. haematobium* infection were followed up until delivery. Additionally, a subsample of women with negative urine samples was recruited as a non-infected control group (1:1 ratio infected and non-infected groups) and followed up until delivery. Only participants with available macerated placental samples were considered for final analysis. Placental samples were subjected to light-microscopy-based screening for *S. haematobium* eggs and PS was considered present if a least one *S. haematobium* egg was detected. Positive light microscopic placental samples were confirmed by qPCR.

**Results:**

Among 318 women screened for *S. haematobium* in urine, we found 40 (12.6%; 95% CI: 9.1-16.7%) to be positive. Together with 40 women in the non-infected control group all women were followed up until delivery. After loss-to-follow-up, 28 (70%; 28/40) women with *S. haematobium* infection and 20 (50%; 20/40) without infection provided placenta samples at delivery. In the group with *S. haematobium* infection, 14% (4/28; 95% CI: 4.0-32.7%) of women were positive for *S. haematobium* eggs in macerated placenta tissue. In the non-infected control group, one woman (5%; 1/20; 95% CI: 0.1-24.9%) had a positive microscopy result for PS. All five women with positive *S. haematobium* egg microscopy in placental tissue received a concordant qPCR result.

**Conclusion:**

14% of women with *S. haematobium* infection also had PS. Notably, PS was also observed in 5% of women without detectable *S. haematobium* eggs in urine. This suggests that PS could be an underestimated phenomenon in highly endemic regions and warrants further investigations of its implications for mother-and-child health.

## Author summary

Schistosomiasis is a neglected disease that affects millions of people, mostly in sub-Saharan Africa. One of the main species, *Schistosoma haematobium*, is known to infect the urinary and genital organs; however, little is known about this infection during pregnancy, and even less is known about whether the parasite can affect the placenta. We collected placental tissue from women with and without *S. haematobium* infection and applied a laboratory method that allowed for the examination of large amounts of tissue. A total of 318 pregnant women participated in this study among whom 40 women tested positive for *S. haematobium* eggs in urine. Those with *S. haematobium* infection and a control group of 40 women without detectable eggs in urine were followed-up until birth. Among the 28 infected participants who could be followed-up until birth eggs were found in the placenta of four women, but also in one woman without detectable infection in urine. These results show that the parasitic infection can involve the pregnant uterus and the placenta. This underlines the need to better understand *S. haematobium* infection during pregnancy and supports ongoing efforts to better understand its implications for mother-and-child health.

## Background

Schistosomiasis is a neglected, tropical, waterborne parasitic disease primarily found in tropical and subtropical regions. Infection occurs when certain parasite stages, called cercariae penetrate the human host’s skin upon contact with infested fresh water after which the parasites mature in the human body and ultimately migrate to specific venous plexuses where eggs are deposited [1,2]. *Schistosoma (S.) mansoni* predominantly migrates to the mesenteric venous plexus causing intestinal schistosomiasis where eggs are excreted in stool [2].

**Fig 1:**
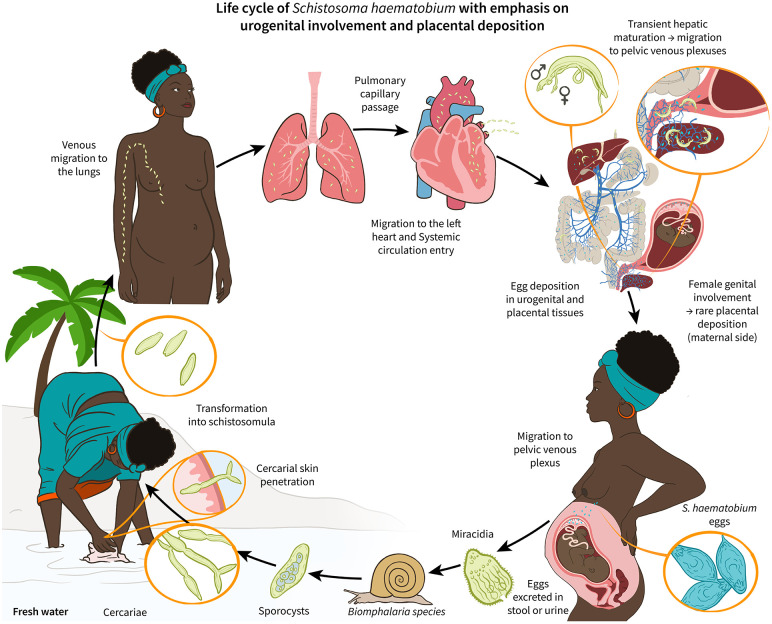
Life cycle of Schistosoma haematobium with emphasis on urogenital involvement. Schematic showing the S. haematobium life cycle in a pregnant host and a hypothesized pathway to placental involvement. After contact with contaminated freshwater, cercariae penetrate the skin, then enter blood vessels to be transported to the heart from where they reach the systemic circulation. Schistosomula mature in the liver, then adult worms typically migrate to pelvic venous plexuses (vesical, uterine, vaginal). Female worms release eggs that either pass into the bladder for excretion via urine or become entrapped in adjacent tissues. In rare cases, eggs may reach the placenta through interconnected venous plexuses of the urogenital tract and therefore, the gravid uterus.

In contrast, *S. haematobium* migrates to the venous plexus of the bladder, leading to urogenital schistosomiasis where eggs are excreted in urine [2]. Beyond the bladder vessels, *S. haematobium* may reach the rectal, uterine, and vaginal venous plexuses, creating potential pathways for egg deposition in the genital tract causing female genital schistosomiasis a frequent ectopic disease manifestation. Ectopic schistosomiasis is a condition of *Schistosoma* eggs deposited and trapped in tissues other than those typically associated with the disease, such as urogenital tract and intestinal tract. Although being a rather rare phenomenon, ectopic schistosomiasis is well documented particularly for *S. haematobium* [3]. The valveless anatomy of the vertebral and pelvic venous plexuses suggests a biologically plausible route for active *S. haematobium* adult migration and egg metabolization towards the uteroplacental blood vessels allowing for entrapment of eggs in placental tissue (Fig 1). Thus, placental schistosomiasis (PS) can be considered a rare manifestation of ectopic schistosomiasis in women infected particularly by *S. haematobium* [4–8]. However, placental involvement in schistosomiasis has long been recognized: the first case of human PS was reported as early as 1948, and the first systematic prevalence study was conducted in the 1970s by Renaud et al. [9,10].

Globally, schistosomiasis remains a public health concern in tropical and subtropical areas, putting 770 million people at risk of infection, with approximately 253.7 million requiring regular preventive drug treatment, of whom 93.9% reside in Africa [11]. Over 3.3 million disability-adjusted life years globally are caused by schistosomiasis annually, and women of reproductive age are among the most affected populations [12–14]. Although praziquantel (PZQ) is safe and recommended by the World Health Organization (WHO) since 2002 for pregnant women, many countries still exclude them from treatment out of considerations of potential embryotoxicity. However, untreated *S. haematobium* infections may contribute to the development or even the aggravation of anaemia and malnutrition. These are factors already independently associated with adverse birth outcomes and egg deposition in the placenta may further induce inflammation at the feto-maternal intersection thereby increasing the risk of adverse birth outcomes at least in theory [9].

Valid and reliable diagnostic tools for PS are lacking. Histopathology has its limitations, as it is feasible to only assess a part of the total sample; this results in poor sensitivity of this diagnostic method [10]. To improve detection, potassium hydroxide (KOH) maceration techniques were developed, which enabled the examination of larger volumes of tissue [10,15]. To date, such methods have not been tested in the field for PS screening.

Thus, our study aimed to assess the diagnostic feasibility of an improved KOH-based tissue maceration technique to detect PS in a highly endemic region for *S. haematobium* in Gabon and to determine the prevalence of PS among pregnant women with light microscopically determined *S. haematobium* infection.

## Methods

### Ethics statement

This study received ethical approval from the Medical Board in Hamburg, Germany (2021–100664-BO-ff) and the Institutional Review Board of the Centre de Recherches Médicales de Lambaréné (CERMEL), Gabon (CEI-009/2021). All participating women signed a written informed consent form before any study-related procedure was performed. The study was conducted according to the International Council for Harmonization of Technical Requirements for Pharmaceuticals for Human Use-Good Clinical Practice principles and the Declaration of Helsinki.

### Study settings and population

This cross-sectional, diagnostic proof-of-concept study was conducted at the CERMEL [16]. The study region is in the tropical rainforest of central Gabon, approximately 100 km south of the capital, Libreville. Previous publications have reported a regional prevalence for *S. haematobium* between 9% to 25% among pregnant women [17,18]. Thus, in this study, our goal was to recruit pregnant women in a highly endemic region for *S. haematobium* in Gabon, to determine the prevalence of PS among pregnant women with light-microscopically determined *S. haematobium* infection in urine, and to assess the diagnostic feasibility of an improved KOH-based tissue maceration technique [15]. This includes all pregnant women positive for *S. haematobium* infection who presented at the antenatal care unit of the Albert Schweitzer Hospital between January 2022 and January 2023, from 08:00–12:00. Pregnant women from the second trimester onwards were invited by the study team to participate and upon provision of written informed consent, they were screened for *S. haematobium* eggs in urine. Infected pregnant women were then followed until delivery for sampling of the placenta. After delivery, all infected women were treated with a 40 mg/kg single dose of PZQ. Additionally, a control group (1:1 ratio) of women negative for *S. haematobium* eggs in urine was followed up until delivery and sampling of placental tissue. For each participant infected with *S. haematobium*, an uninfected participant recruited during the same week was randomly selected for the control group. Then, macerated placenta samples were screened for the presence of *S. haematobium* eggs in both groups.

### Detection of maternal *S. haematobium* infection in urine and of placental schistosomiasis

Maternal *S. haematobium* infection was determined via light microscopical assessment of a 10 mL urine sample following a widely adopted diagnostic standard in endemic countries [19]. Participants were considered infected if at least one *S. haematobium* egg was detected in urine. Presence of *S. haematobium* eggs detected in macerated placental tissue samples (full-thickness bioptic placenta sections) was assessed using an improved KOH-based tissue maceration technique [15]. Placental tissue was obtained after delivery and dissected into five full-thickness sections of each approximately 5 x 5 cm, according to the Standard Operating Procedure: one central (near cord insertion) and four peripheral (placental edge) samples. As only selected sections rather than the entire placenta were processed, a reduction in sensitivity remains possible if egg deposition is spatially focal. However, the standardized sampling of five spatially distinct full-thickness sections was intended to minimize this potential limitation and capture potential heterogeneity of egg distribution in tissue across samples. Free membranes were not sampled. Immediately after delivery, the placenta was rinsed with water, positioned with the foetal surface facing up, and full-thickness sections, including the maternal surface, were obtained by fresh incisions (no surface scrapings). Each section was placed into a 50 mL jar with 70% ethanol, labeled and stored at -20 °C. Single-use scalpels were discarded in sharps containers; flat surfaces and wash basins were cleaned with bleach between cases. Once ready to be processed, each sample was divided into smaller pieces (about 1 x 1 cm) and placed into 50 mL tubes. 4% KOH was added to each tube until a final volume of 45 mL was reached. Tubes were gently shaken to ensure the placental samples were fully immersed in the KOH solution and then incubated at 37°C in a warming cabinet for 24 hours, with the caps loosely placed on the tubes to allow gas release. After 24 hours, the tubes were inspected to assess whether tissue maceration succeeded. Once maceration succeeded, the tubes were centrifuged for 10 minutes at 2,500 rpm at room temperature leading to the creation of a diagnostic pellet [15]. The diagnostic placenta pellets were then examined for eggs of *S. haematobium* using light microscopy at 100x and 400x magnification [15]. The whole pellet from the macerated placenta tissue was examined by light microscopy. All material was mounted and read as described (no subsampling). Placentas were classified as positive for PS in the case of presence of at least one schistosome egg.

### DNA isolation from KOH-Macerated placental tissue

All microscopically positive macerated placenta samples were further subjected to confirmatory analysis by qPCR. DNA was extracted from KOH-macerated placental samples using the Qiagen DNeasy Blood and Tissue Kit. A 500 µL aliquot of placental tissue was washed twice with 1 mL sterile phosphate-buffered saline. The pellet was resuspended in 180 µL ATL buffer and 20 µL Proteinase K, followed by overnight incubation at 56°C in a thermomixer. DNA was purified according to the manufacturer’s protocol and eluted in 200 µL AE buffer. For molecular detection of *S. haematobium* we followed the protocol published by Cnops et al., focusing on the Dra1 target [20].

### Outcomes

The aim of this study was not to estimate the overall prevalence of PS in the target region, but to estimate the prevalence of PS among all infected women and in a sub-sample of all non-infected women (assuming that the urine screening test would be false-negative in some women). However, in the interest of comparability of our findings to findings in other settings we computed a hypothetical overall PS prevalence via an extrapolation method. First, we used the percentage of PS among the sub-sample of non-infected women to ascertain the hypothetical number of women with PS among all recruited non-infected women (# PS_non-infected group_). Then, accounting for probable losses to follow-up in the infected group, we would use the percentage of PS among the sample of infected women to ascertain the hypothetical number of women with PS among all recruited infected women (# PS_infected group_). Finally, we combined the hypothetical numbers of PS from non-infected and infected groups and divided it by the total number of recruited pregnant women to approximate the hypothetical PS prevalence in the target region ([# PS_non-infected group_ + # PS_infected group_] / # of recruited pregnant women).

### Statistical methods

Data were recorded on paper-based source documents and transcribed to an electronic database (Redcap, Vanderbilt University, USA). Statistical analysis was performed using STATA 17.0 Basic Edition (StataCorp LLC, TX, USA). The baseline characteristics of all women were presented using descriptive statistics. Continuous data were summarized and presented in median (interquartile range [IQR] and ranges) in case of non-parametric data distribution. Categorical data were presented as absolute numbers and relative percentages. 95% confidence intervals (CIs) were constructed for the proportion of women with PS. Spearman’s rank correlation coefficient was calculated to determine correlations between egg count in matched samples of macerated placenta and maternal urine.

## Results

### Prevalence of maternal infection with *S. haematobium* and placental schistosomiasis

**Fig 2:**
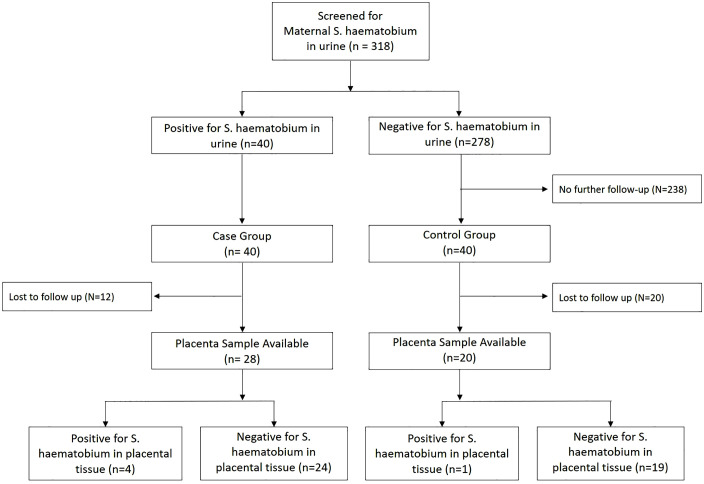
Study flow chart. Flow diagram of screening, group allocation, follow-up, and placental results. Of 318 pregnant women screened by urine microscopy, 40 were positive for S. haematobium (infected group) and 278 were negative. A subset of negatives (n = 40) formed the non-infected control group; the remaining negatives (n = 238) were not followed up further. Placental tissue was available at delivery from 28 infected and 20 control participants (lost to follow-up: n = 12 and n = 20, respectively). Placental schistosomiasis—defined as the presence of S. haematobium eggs in placental tissue—was detected in 4/28 infected participants and 1/20 controls; the remainder were negative (24/28 and 19/20).

Among the total of 318 pregnant women screened for *S. haematobium* in urine, infection was detected in 12.6% (40/318; 95% CI: 9.1-16.7%). All 40 women with confirmed *S. haematobium* infection were followed up until delivery (Fig 2). However, 30% (12/40) among them were lost to follow-up due to delivery outside of the study site, leaving 28 (70%; 28/40) with available placental samples. From the non-infected control group, 50% (20/40) were lost to follow-up, due to the same reason, resulting in 20 available samples. Of 48 deliveries, two were by caesarean section; PS was identified in one of these two cases. Given the sterile operative field and routine bladder catheterisation during caesarean delivery, urine contamination of the placenta is unlikely. Among infected women with available placental samples, the prevalence of PS was 14.3% (4/28; 95% CI: 4.0-32.7%), whereas among the women from the non-infected, control group with available placental samples the prevalence of PS was 5% (1/20; 95% CI: 0.1-24.9%). Assuming that the 5%-PS-prevalence among the sub-sample of non-infected women extends to all 278 non-infected women, it would mean that there may have been a total of 14 women (95% CI: 0–70) with PS among them. Similarly, assuming that the 14%-PS-prevalence among the sample of infected women extends to all 40 recruited infected women, it would mean that there may have been a total of six women (95% CI: 2–13) with PS among them. Combining these hypothetical and extrapolated numbers of PS, the overall hypothetical PS prevalence among pregnant women in the target area could be as high as 6.3% (20/318; 95% CI: 3.9-9.5%). All placental samples of women with detectable PS showed concordant qPCR results. For one sample, the whole diagnostic pellet was used in light microscopy, so there was insufficient material for qPCR assessment.

### Participant characteristics

There was no relevant difference in age between women with *S. haematobium* infection in urine and those without infection (median 25 years [IQR: 21 – 31] vs. 26 years [IQR: 20 – 34]; p = 0.76) as shown in Table 1. Baseline characteristics of women who completed and those who did not complete the study are summarised in S1 Table.

**Table 1:** Maternal S. haematobium infection as defined by presence of eggs in urine cross-tabulated by placental schistosomiasis and age.

Characteristics	Infection with *S. haematobium*	
	Positive (n = 28)	Negative (n = 20)
Age		
Median age in years (IQR)	25 (21 – 31)	26 (20 – 34)
Placental schistosomiasis		
Positive	4 (14.29%)	1 (5%)
Negative	24 (85.71%)	19 (95%)

### Infection intensity

Among women with *S. haematobium* infection, the egg count in urine ranged from 1 to 870 eggs per 10 mL. The median egg count was 50 (IQR: 8 – 113). The number of *S. haematobium* eggs detected in placental tissue ranged from 2 to 36 eggs per examined placental sample (Table 2). Spearman’s rank correlation coefficient indicated a positive correlation between placental egg count and maternal egg count (Spearman’s rho = 0.72). However, even if this dose-response effect is biologically plausible it should be interpreted with caution since it might be due to sampling variation (p = 0.17) owing to the very small sample size (n = 5) in this analysis.

**Table 2:** Number of eggs detected in macerated placental tissue samples (full-thickness bioptic placenta sections) and maternal urine among the five women with placental schistosomiasis.

	*S. haematobium* (eggs in macerated placental tissue samples)	*S. haematobium* (eggs per 10 mL of urine)
Participant 1	2	48
Participant 2	4	27
Participant 3	6	870
Participant 4	36	200
Participant 5	2	0

## Discussion

This study is the first observational study to determine the prevalence of PS using the improved maceration technique in a highly endemic region for *S. haematobium* in Gabon. Overall, we found a prevalence of *S. haematobium* infection of 12.6% (40/318; 95% CI: 9.1-16.7%) among pregnant women, among whom the prevalence of PS was 14.3% (4/28; 95% CI: 4.0-32.7%). Interestingly, there was also one positive case of PS (5%; 1/20; 95% CI: 0.1-24.9%) among women without *S. haematobium* infection. These findings confirm that placental involvement seems to be a common phenomenon among pregnant women with *S. haematobium* infection. This high percentage occurs in the context of low levels of schistosomiasis care that pregnant women and their offspring receive in endemic settings, suggesting that they constitute an overlooked population within the already neglected field of schistosomiasis.

### Epidemiology of urinary and placental schistosomiasis

PS has been previously documented only sporadically, mostly in case reports or case series [9]. To date, only a systematic study investigating PS, Renaud et al. identified a PS prevalence of 22.6% (73/322) among pregnant women in Ivory Coast in 1972 using a placenta maceration technique and light microscopy [10]. In a recent study, Franz et al. confirmed these results, reporting a prevalence of 19% (51/268) among placental samples from women in the Ivory Coast and Ghana using qPCR to detect *Schistosoma* spp. [21]. Both studies assessed placental samples for schistosomal infection among women with unknown maternal infection status. In contrast, our study design was a cross-sectional study investigating PS among women with microscopically confirmed *S. haematobium* infection as well as in an uninfected control group. The estimated hypothetical overall PS prevalence was 6.3% (95% CI: 3.9-9.5%) and was therefore noticeably lower compared to the reported results from Franz et al. and Renaud et al. One explanation for this lower PS prevalence could be that the *S. haematobium* prevalence in the studies conducted by Renaud et al. and Franz et al. had a higher overall prevalence of maternal schistosomiasis in their study samples of pregnant women. Another explanation would be that infection intensity was higher in these other two studies. However, since neither of these studies reported maternal *S. haematobium* infection prevalence or infection intensity, this remains speculative. Lastly, we used an extrapolation method to estimate the hypothetical PS prevalence, assuming that the non-infected women and the infected women were representative of all non-infected and infected women, respectively, in the target region. However, this might not be the case, and therefore it is equally possible that this estimation constitutes an underestimation, but also an overestimation.

Meanwhile, the prevalence of maternal *S. haematobium* infection in the present study is in good agreement with other previously reported figures. In a recent review and meta-analysis, Adam et al. reported an estimated prevalence of maternal *S. haematobium* infection of 21.1% (95% CI: 14.1 – 28.1) [22]. Previous publications from the same study region as the present study have reported similar figures. The cross-sectional study from Mombo-Ngoma et al. found a prevalence of 9% (103/1.115) among pregnant women between September 2009 and November 2013, while in a more recent randomized controlled trial Gerstenberg et al. reported a prevalence of 25.1% among pregnant women recruited from 2019 – 2023 in the same area [17,18]. Differences between reported prevalences may have been caused by differing sampling strategies and different diagnostic approaches. While Mombo-Ngoma have used only one single urine sample assessed by urine filtration and light microscopy, Gerstenberg et al. have collected urine samples on three consecutive days, and diagnostics have also included analysis of circulating anodic antigen for the diagnosis of *S. haematobium* infection.

### Diagnostic approach

Conventional histopathology has limited diagnostic sensitivity because only small tissue fragments can be examined due to feasibility aspects. The improved KOH-based tissue maceration technique addresses these limitations by allowing efficient examination of larger placental tissue volumes, thereby increasing the likelihood of detecting eggs [15]. Compared to earlier maceration methods where the egg shape was often destroyed and the spine was often obscured, the improved KOH-based tissue maceration technique better preserves egg morphology, allowing for better species identification. Additionally, using disposable 50 mL tubes and cutting placental tissue into multiple small samples of 1 x 1 cm before maceration lead to a larger surface area exposed to the KOH solution, promoting more effective tissue maceration and consequent egg release. Also, the improved KOH-based tissue maceration technique did not require extensive training, was easy and quick to use for laboratory technicians [15]. Meanwhile, this study is the first one to apply this improved maceration technique investigating PS among pregnant women with *S. haematobium* infection in real-world settings.

### Placental schistosomiasis and adverse birth outcomes

Schistosomiasis has been linked to adverse birth outcomes such as prematurity, low birth weight and stillbirth, but underlying mechanisms are not yet fully understood [23]. Mombo-Ngoma et al. carried out a study in the same region in 2017 and found an increased risk for low birth weight (aOR: 1.93 [95% CI: 1.08–3.42], p = 0.04) among mothers infected with *S. haematobium* (n = 103/1115) [17]. However, this was not consistent with two other studies: In 2019, Murenjekwa et al. investigated low birth weight among pregnant women in rural Zimbabwe, who were infected with *S. haematobium* (n = 471/4437), and found no significant risk (aOR: 1.26 [95% CI: 0.85-1.86], p = 0.26) [24]. In 2021, Honkpehedji et al. found an increased risk for low birth weight (OR: 1.94 [95% CI: 1.28-2.91], p = 0.002) among women infected within *S. haematobium* in Gabon (n = 47/142), but statistical significance was not persistent after correcting for confounders in multivariable analysis (aOR: 1.57 [0.96-2.57, p = 0.07] [25]. However, none of these studies investigated the association of adverse birth outcomes with PS, leaving an important gap in understanding potential contribution of PS to these complications.

**Fig 3:**
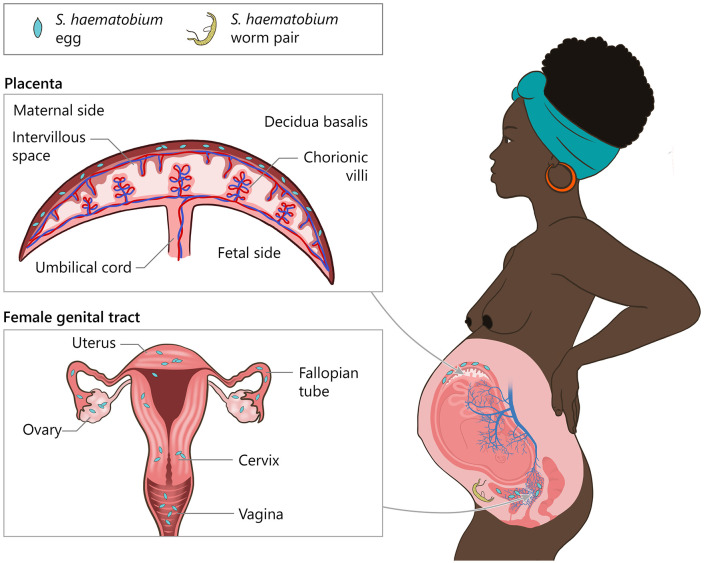
Female genital tract and placental schistosomiasis. *Adult S. haematobium pairs reside within pelvic venous plexuses (vesical, uterine, vaginal). This schematic is intended to support anatomic plausibility and does not imply a single proven route of egg migration. Schematic of the female genital tract and placenta highlighting anatomic sites affected by urogenital schistosomiasis due to S. haematobium. Eggs may get entrapped in the lower genital tract (vulva, vagina, cervix) and the upper tract (endometrium/myometrium, fallopian tubes, ovaries). The placenta panel depicts the maternal and fetal sides of the placenta, with the intervillous space; S. haematobium eggs may be found on the maternal side*.

PS has been described in infections both with *S. haematobium* and *S. mansoni*, although when considering the number of cases, *S. haematobium* was far more frequently reported [9]. *S. haematobium* primarily targets the urogenital tract, and thus, ectopic schistosomiasis in the placenta may be more frequent, as the placenta is embedded within the venous plexuses of the reproductive organs. We suggest the interconnectedness of the uterine, vaginal, and vesical venous plexuses may provide a potential route for *S. haematobium* eggs to reach the placental tissue via embolization and active migration of the adult parasite [2]. Additionally, it may be possible that *S. haematobium* may migrate directly to the vessels surrounding the placenta and may deposit eggs in the placenta. However, the pelvic blood flow is increased during pregnancy, which might favor higher rates of passive egg transportation via the venae uterinae to the placenta (Fig 3), also facilitating manifestations of PS in intestinal schistosomiasis [2].

Most of the case reports and case series describing PS are reported from women with adverse birth outcomes, and the authors have often suggested that PS may have been associated with those [9]. However, such publications have a high risk for selection bias, since positive or particularly salient findings have a higher likelihood of being published. So far, the study from Renaud et al. was the only one that investigated the association between PS and adverse birth outcomes systematically. Renaud et al. found no difference in birthweight, height or proportions of low birth weight between newborns from mothers with PS compared to those without PS. Additionally, the authors reported that PS was significantly more common among cases of spontaneous abortion occurring during the second trimester than among spontaneous abortions occurring during the first trimester (32.1% vs 2.6%, p = 0.01). The authors concluded that PS might generally manifest during the second and third trimesters [10]. Evidence for adverse pregnancy outcomes linked to *S. haematobium* is limited; our findings pertain to placental egg detection rather than clinical effects.

### Possible mechanisms of Placental Schistosomiasis contributing to adverse birth outcomes

Mechanisms through which maternal schistosomiasis contribute to adverse birth outcomes remain constantly under debate. Mechanisms include maternal anemia, peripheral inflammation and malnutrition, as well as placental and fetal inflammation [23,26]. The first three mechanisms represent symptoms of maternal schistosomiasis, which can occur independent of placental involvement. Especially maternal anemia has been well studied in schistosomiasis and is linked to adverse birth outcomes [22,27–29]. On the other hand, placental and fetal inflammation represent mechanisms closely related to PS and may be driven by transplacental transfer of antigens and placental egg deposition. In maternal schistosomiasis, soluble egg antigens (SEA) frequently show transplacental passage—reported in up to 86% [30]. In a human placental in-vitro model, SEA elicited a dose-dependent increase in pro-inflammatory cytokines [30–33]. These observations support biological plausibility for local placental inflammation even when intact eggs are not histologically detected [30–33]. A healthy placenta is usually characterized by a cytokine profile linked to Th-2 cells. A shift towards proinflammatory cytokines has been associated with adverse birth outcomes both in schistosomiasis as well as other parasitic diseases [14,34,35]. Further studies investigating immunologic profiles in individuals with PS might therefore further elucidate the role of PS in the development of adverse birth outcomes during maternal schistosomiasis.

### Treatment recommendation & policy implications

PZQ has been the only available licensed drug for treating schistosomiasis since its market introduction in 1972 and has been withheld from pregnant women for decades due to concerns of potential embryotoxicity [36]. Only in 2002, the WHO changed its recommendations, now supporting the use of PZQ in pregnant women in both individual treatment strategies as well as in mass drug administration programs [37]. Since then, three randomized controlled trials have investigated the safety and anti-schistosomal efficacy of PZQ in pregnant women, each one targeting one of the three main species responsible for human schistosomiasis (*S. mansoni, S. japonicum and S. haematobium*) [18,38,39]. While all three trials have shown a good safety profile of PZQ during pregnancy (2^nd^ and 3^rd^ trimesters), the evidence for a beneficial effect on the current pregnancy remains under debate. The recommendation for the treatment of maternal schistosomiasis during pregnancy is currently based on a risk-benefit consideration that takes into account potentially beneficial long-term effects of the therapy and the situation of medical care in endemic countries and low-resource settings. While to date, neither risks nor immediate beneficial effects of a treatment on the outcome of the current pregnancy have been demonstrated [18,38,39], the existing preliminary evidence still suggests that maternal schistosomiasis is probably unfavorable for the offspring [17,23,25,26]. Furthermore, withholding treatment upon diagnosis during pregnancy can lead to a significant delay in treatment, particularly in low-resource settings, thus resulting in increased morbidity. Therefore, it is recommended that treatment is given in such circumstances [18,36,40]. Antenatal care presents a unique time when women interface with the health care system, and if not addressed during pregnancy, may be missed in mass drug administration campaigns for many reasons, including school-based approaches and missed opportunities during community-based interventions. An additional aspect in favor of treatment during pregnancy is a reduction in maternal clinical symptoms [18]. PS, in turn, is considered a complication of maternal schistosomiasis, which develops during the course of pregnancy, and authors have suggested that PS manifests from the 3^rd^ month of gestation onwards [10]. If proven that the risk of adverse birth outcomes is particularly high in women with PS, this would further demonstrate important evidence in favor of early treatment of schistosomiasis during pregnancy.

However, the question remains whether PZQ treatment should be implemented in highly endemic settings as part of test-and-treat strategies or as part of mass drug administration programs. This question depends largely on the local prevalence and the sensitivity of the applied screening methods. To date, it is common practice in endemic countries to screen a single sample of 10 mL of urine for *S. haematobium* egg detection [19]. However, light microscopic assessment of a single urine sample of 10 mL has limited diagnostic sensitivity. In this light it is important to highlight the current WHO guidelines in which it is recommended to include pregnant women (after the first trimester) in preventive PZQ chemotherapy programs in endemic areas.

### Strengths & limitations

This is the first study to evaluate PS using the improved maceration technique in real world settings in a highly endemic region of Gabon. In contrast to the so far only study systematically investigating PS from Renaud in 1972, we also investigated the infection status of the mother via urine light microscopy, making it possible to compare maternal and PS prevalences within the same study sample. Meanwhile, a high number of dropouts lead to a decreased sample size of our study. However, it was possible to ascertain data on basic demographic characteristics on women who completed the study and those who did not complete the study (S1 Table). Since the distribution of this data did not significantly differ between the two groups it is likely that overall results would have been similar even if all participants had been followed up until birth. Morphological characteristics of *S. haematobium* eggs were well visible in all cases of PS, and confirmatory qPCR analysis corroborated these results. However, we concede that the small sample size has affected the precision of this study as demonstrated by the wide 95% CIs around the PS point estimates. Another limitation related to the low sample size constitutes the fact that we could not assess any potential association between PS and adverse birth outcomes. While our study ascertained data to investigate this association, we concluded that due to data sparsity, no meaningful inferences can be made from the results of this study. Instead, future larger studies should be conducted for such an investigation. Furthermore, urine screening for *S. haematobium* was only done on one day, while screening on three consecutive days could have increased the diagnostic sensitivity. This is even demonstrated by our findings since there was one case of PS in a woman with a negative light microscopic result. However, it was not the objective of this study to categorize women by infection status using diagnostic gold standard methodology, but rather by applying common diagnostic algorithms in endemic settings and discussing the implications of these methods for PS and potential PS treatment campaigns. Lastly, future studies on maternal schistosomiasis should ideally contain placental sampling for evaluation of PS, ascertainment of adverse birth outcomes, immunological assessments and randomized treatment strategies to clarify the unresolved relation between PS and maternal and neonatal health. Addressing these questions will be essential for refining schistosomiasis treatment strategies in pregnancy and informing policymakers on the potential role of PZQ in mitigating adverse birth outcomes.

## Conclusion

Approximately 15% of women with *S. haematobium* infection detected in urine also had PS. Notably, PS was also observed in 5% of women without detectable *S. haematobium* eggs in urine. This suggests that PS could be an underestimated phenomenon in highly endemic regions and warrants further investigations of its implications for mother-and-child health. Most importantly, future research should assess whether PS is associated with adverse birth outcomes or negative health consequences for the offspring. The findings in this study highlight that pregnant women and their offspring remain an overlooked population within the already neglected field of schistosomiasis.

## Supporting information

S1 Data Dataset used for statistical analysis. (XLS)

S1 Table Characteristics of study participants who completed the study and those who did not complete the study. N.b. Among non-completers data was fully available only on age and gravidity. For other variables results are displayed based on available data.
(DOCX)
