# Supplementary material for: The underestimated burden of placental schistosomiasis in endemic regions: Findings from a cross-sectional, diagnostic proof-of-concept study on placental schistosomiasis in Gabon
Source: PLoS Negl Trop Dis. 2026 Mar 3;20(3):e0014031. doi: 10.1371/journal.pntd.0014031 (PMC13001911; doi:10.1371/journal.pntd.0014031)
Supplement: S1 Table — N.b. Among non-completers data was fully available only on age and gravidity. For other variables results are displayed based on available data. (DOCX) [file pntd.0014031.s002.docx]

S1 Table: Characteristics of study participants who completed the study and those ones who did not complete the study. N.b. Among non-completers data was fully available only on age and gravidity. For other variables results are displayed based on available data.

| **Variable** | **Non-completers**  **(n=32)** | **Completers**  **(n=48)** | **p-value** | **Test** |
| --- | --- | --- | --- | --- |
| **Age (n, IQR) years** | 24 (IQR: 22 – 30) | 25 (21 – 31) | 0.966 | Wilcoxon ranksum test |
| **Gravidity (n, SD) years** | 2.94 ± 1.98 | 3.02 ± 2.09 | 0.926 | Wilcoxon ranksum test |
| **HIV positive (n, %)** |  |  |  |  |
| **Positive** | 5 (31.25%) | 9 (21.43%) | 0.500 | Fisher’s exact test |
| **Negative** | 11 (68.75%) | 33 (78.57%) |  |  |
| **Illiterate (n, %)** |  |  |  |  |
| **Yes** | 25 (100.00%) | 45 (95.74%) | 0.540 | Fisher’s exact test |
| **No** | 0 (0.00%) | 2 (4.26%) |  |  |
| **Smoking during pregnancy (n, %)** |  |  |  |  |
| **Yes** | 0 (0.00%) | 0 (0.00%) | 1.0 | χ² test |
| **No** | 17 (100.00%) | 45 (100.58%) |  |  |
| **Alcohol consumption during pregnancy (n, %)** |  |  |  |  |
| **Yes** | 3 (17.65%) | 10 (22.83%) | 0.750 | χ² test |
| **No** | 14 (82.35%) | 34 (77.27%) |  |  |
| **Education level (n, %)** |  |  |  |  |
| **No education level** | 0 (0.00%) | 1 (2.22%) | 0.707 | χ² test |
| **Primary school completed** | 4 (16.00%) | 9 (20.00%) |  |  |
| **Secondary school completed** | 21 (84.00%) | 34 (75.56%) |  |  |
| **Higher education completed** | 0 (0.00%) | 1 (2.22% |  |  |
| **Main income (n, %)** |  |  |  |  |
| **Full time employment** | 0 (0.00%) | 1 (2.08%) | 0.617 | χ² test |
| **Self-employment** | 2 (8.00%) | 2 (4.17%) |  |  |
| **No occupation** | 23 (92.00%) | 45 (93.75%) |  |  |
